# Is Cancer History Related to Neurologic Specialty Care in Patients with Dementia?

**DOI:** 10.1093/geroni/igab046.2329

**Published:** 2021-12-17

**Authors:** Mackenzie Fowler, Kristen Triebel, Gabrielle Rocque, Ryan Irvin, Richard Kennedy, Nicole Wright

**Affiliations:** University of Alabama at Birmingham, Birmingham, Alabama, United States

## Abstract

Background: The incidence and prevalence of aging-related diseases such as dementia and cancer are increasing, as are cancer survival rates. Cancer and its treatments have been associated with cognitive effects for those who later develop dementia. Guidelines have suggested that cancer patients return to follow-up in primary care following remission and be referred to specialists for cognitive complications, but it is unclear how well these guidelines are followed. Methods: Electronic health record data at the University of Alabama at Birmingham were extracted from July 2003 May 2020. Rates of specialty care utilization on or after dementia diagnosis were compared by cancer history status in adults 50 years old or older at dementia diagnosis. Predictors of specialty care utilization were examined using logistic regression. Results: Rate of specialty care utilization was lower for those with cancer history compared to those without on the date of dementia diagnosis (11.3% vs. 17.1%) and after diagnosis (13.5% vs. 19.2%). Older age at dementia diagnosis, non-Hispanic Black race, anticholinergic burden, socioeconomic status, and vascular risk factors were associated with lower odds of specialty care utilization. Dementia medication use was associated with higher odds of specialty care utilization on and after dementia diagnosis. Conclusions: Cancer survivors with a dementia diagnosis are less likely to utilize specialty care than those with no history of cancer. Several factors predicted specialty care utilization. Additional studies should assess potential barriers in referring cancer survivors to specialty care for cognitive impairment.

